# Anæsthetic in New Form

**Published:** 1876-03

**Authors:** George Halsted Boyland

**Affiliations:** Baltimore, Md.


AN ANÆSTHETIC IN A NEW FORM—INTRAVENOUS INJECTION OF HYDRATE OF CHLORAL—*By George Halsted Boyland, M. D., M. A., etc., Baltimore, Md.*—Chloroform and sulphuric ether boast of such general usage that it might provoke a smile to lose many words about another anæsthetic. Nevertheless, every one knows that the exhibition of these drugs is by no means without danger; therefore we ought certainly to accept with pleasure a harmless substitution in its place.

Such an one appears to be the hydrate of chloral in its latest method of administration, namely: in the form of injections into the veins. Cuvelier, Battalion-Surgeon, describes, in the *Archives Medicales Belges*, this new procedure in the following manner: The method of injecting medicaments of different kinds into the veins is by no means new. It was already used at the time of the Alexandrian school; was soon, however, quite forgotten, until, through Ore, Professor of Physiology at Bordeaux, it came again into vogue. He employed now chloral only; in order to have a means that, for major surgical operations, could produce complete anæsthesia for a long time. Although this savant obtained by his experiments only good results, this method of giving chloral did not receive the sympathy of either the Academie de Medicine or the Societie de Chirurge, of Paris. Deneffe and Van Wether, who repeated the experiments of Ore, came also to the opinion that the chloral injected into the veins was a powerful anæsthetic, and, by much, preferable to chloroform.

The opposers of this new method brought forward that through it the formation of small coagula which cause emboli was favored; also, that inflammation of the veins, as well as entrance of air into the same, could very easily transpire; and mentioned several other objections. But the facts give a brilliant dementi to these quasi evils: *In* 22 *cases in which the intra-venous injections of chloral have been used, we count* 22 *successes.*

The two above named experimenters have laid before the Society of Physicians of Gand a memoir in this connection, the closing phrases of which run as follows: "The question has been proposed to us, whether the anæsthesia brought about by injections into the veins be preferable to that produced by inhalation. We answer this question to the effect that the first procedure—

1. Works surer and quicker. Complete anæsthesia is produced in the shortest time—6 to 10 minutes.

2. This insensibility is mathematically gauged, and, therefore,

3. The duration of the same depends upon the size of the injected dose.

4. Never is nausea or vomiting produced, even when the subjects come from the table.

5. Sleep and anæsthesia are occasioned *without a primary stage of excitation*, which, during the administration of chloroform or other anæsthetics, always takes place. Finally, with reference to the *harmlessness* of this method, it is thus far perfect; not the slightest accident before or after the injection has, as yet, been observed; the same cannot be affirmed of chloroform.

The hydrate of chloral should only come into use as a purely chemical, always an *ex tempore* preparation—for the reason that it decomposes very readily, and then can work in the most harmful manner.

Having prefaced the above, we now mention that Profs. V. Deneffe and A. Van Wether, of the Gand University, have already published at Brussels a long brochure upon this new method. Their work embraced 34 cases, in which, by means of the intravenous injection of chloral, perfect anæsthesia has been accomplished. In all, this procedure has already been employed in 65 cases; *once*, in consequence of syncope, there was a fatal termination; in 4 cases hæmaturia or albuminuria was present.

To the just cited advantages of this method, the authors add this: that the chloral-sleep lasts from one to two days after the injection and operation, and that, therefore, the sensibility first returns at a time when the patient has long since recovered from the operation shock. Moreover, the insensibility can, without danger, be pushed to an absolute point, so that the cornea does not react upon touch, thus rendering operations on and in the eye much easier to perform.

Although the one case of death casts a shadow upon our method, nevertheless, it is probable that the same will find a place in, and prove to be advantageous to our surgical practice, if the indications and contra-indications, are precisely fixed, and the instruments bettered and rendered more simple.

It is enough for us in this place to have called the attention of the medical world to an anæsthetic in a new form. Perhaps men will be found to try the new method upon animals, and also upon the human subject, and to give their good or bad results publicly for the benefit of mankind.

